# Self-Expressive Creativity in the Adolescent Digital Domain: Personality, Self-Esteem, and Emotions

**DOI:** 10.3390/ijerph16224527

**Published:** 2019-11-15

**Authors:** María del Carmen Pérez-Fuentes, María del Mar Molero Jurado, José Jesús Gázquez Linares, Nieves Fátima Oropesa Ruiz, María del Mar Simón Márquez, Mahia Saracostti

**Affiliations:** 1 Department of Psychology, University of Almería, 04120 Almería, Spain; mpf421@ual.es (M.d.C.P.-F.); foropesa@ual.es (N.F.O.R.); msm112@ual.es (M.d.M.S.M.); 2 Department of Psychology, Universidad Politécnica y Artística del Paraguay, 1628 Asunción, Paraguay; 3 Department of Psychology, Universidad Autónoma de Chile, 4780000 Santiago, Chile; jlinares@ual.es; 4 Escuela de Trabajo Social, Facultad de Ciencias Sociales, Universidad de Valparaíso, 2653 Valparaíso, Chile; mahia.saracostti@ufrontera.cl

**Keywords:** digital domain, self-expressive creativity, personality, self-esteem, emotional intelligence, adolescence

## Abstract

Background: Although self-expressive creativity is related to cyberbullying, it can also reinforce strengths that contribute to positive adolescent development. Our study concentrated on the relationships between personality traits and self-expressive creativity in the digital domain in an adolescent population. For this, we analyzed the effect of self-esteem and emotional intelligence as assets for positive development related to personality traits and self-expressive creativity. Methods: The study population included a total of 742 adolescents that were high-school students in the province of Almería, Spain. The following instruments were used: Big Five Inventory (BFI) to evaluate the five broad personality factors, Rosenberg Self-Esteem Scale (RSE), Expression, Management, and Emotion Recognition Evaluation Scale (TMMS-24), and the Creative Behavior Questionnaire: Digital (CBQD). Results: The cluster analysis revealed the existence of two profiles of adolescents based on their personality traits. The analysis showed that the group with the highest levels of extraversion and openness to experience and lowest levels of neuroticism were those who showed the highest scores in self-esteem, clarity, and emotional repair, as well as in self-expressive creativity. Higher scores in neuroticism and lower scores in extraversion and openness to experience showed a direct negative effect on self-expressive creativity and indirect effect through self-esteem and emotional attention, which acted as mediators in series. Conclusions: To counteract certain characteristics that increase adolescents’ vulnerability to social network bullying, a plan must be developed for adequate positive use of the Internet from a creative model that enables digital self-expression for acquiring identity and self-efficacy through the positive influence of peers, which promotes feelings of empowerment and self-affirmation through constructive tasks that reinforce self-esteem and emotional intelligence.

## 1. Introduction

### 1.1. Self-Expressive Creativity in the Digital Domain and Personality

Cyberbullying refers to the use of communication media (Internet, cell phones, websites, and/or online video games) to exert violence against other persons by publishing images, insult, videos, memes, or any other type of private information that can harm someone [1]. The adolescent attitude has a significant impact on bullying behavior [2,3], and the peer group and emotional intelligence (EI) also play relevant roles [4,5]. Most cyber victims are also in-person victims [6], and women experience the most indirect verbal aggression [7]. 

Despite the risks associated with information and communication technology (ICT), adolescents now socialize through the use of social networks and the Internet. Thus, relationships are often forged and consolidated through the use of technological media [8]. Proof of this is the time that adolescents spend connected to the network using new technologies, mainly to play video games, chat with friends, download or listen to music, and even to create their own virtual content [9]. This everyday use that characterizes these technologies also enables their possible use for creative purposes.

There are as many different definitions of creativity as there are theoretical approaches from which they have been studied. All these definitions are equally valid, and above all, complementary in approaching the same subject of study from different perspectives. Guifford [10] suggested that creative people show divergent thinking, characterized by fluidity, flexibility, and originality in problem-solving. Torrance [11] defined creativity as a process through which people are sensitive to gaps in knowledge to which they attempt to find solutions, formulating hypotheses. For Amabile [12], creativity makes it possible for people to solve problems through their ability to combine ideas which they already have in a different way; thus, creativity involves inventing and discovering, and must provide an appropriate useful solution for experts in that subject. Csikszentmihalyi [13] considered creative people in general to be divergent thinkers, perseverant with their achievements, who incorporate activity followed by reflection in their work dynamics. According to Sternberg and Lubart [14], creativity requires the confluence of six different, but interrelated resources: intellectual abilities, knowledge, thinking styles, personality, motivation, and environment. De Bono [15], however, considered creativity a skill which anyone can learn, practice, and use. His method consists of developing lateral thinking to contribute creative solutions. 

In the literature above, some studies analyze creativity in domains linked to the arts and sciences (music, dance, painting, sculpture, literature, mathematics, physics, etc.) [13,16,17], while others focus on everyday life [18,19]. Everyday creativity refers to self-expression in daily activities, to interpersonal style, to professional activities and problem-solving in everyday life [11,18]. Ivcevic [20] compared both types of creativity (artistic and everyday) and found that they are moderately correlated, which means that both types of creativity showed similarities and differences. On one hand, they both have similar underlying psychological processes, and on the other, while artistic creativity requires acquisition of certain socially recognized skills, everyday creativity is mainly private and does not require the acquisition of specific skills. 

Experts in creativity have paid less attention to analysis of creativity in everyday life, which involves humor and self-expression in daily activities, in correlation with personality traits, such as extraversion and consciousness, as well as personal growth, which is a component of psychological wellbeing [20,21]. Self-expressive creativity in the digital domain, according to Ivcevic and Mayer [21], includes behaviors that express identity and personal interests, handcraft activities, and social expression, and are included in daily life. In this context, one group of researchers found that self-expressive creativity is correlated with personality traits, such as openness to experience [21,22,23,24] and nonconventionality [24]. Personality is constructed from experience based on a network of temperament traits that tend to remain stable over time [25]. We focused on the relationships between personality traits and self-expressive creativity in the digital domain in an adolescent population. In this respect, it should be considered that the study of self-expressive creativity in the digital domain is a relatively new line of research and that, therefore, there are very few studies in the field.

### 1.2. Self-Expressive Creativity in the Digital Domain, Self-Esteem, and Emotions

Adolescents are involved in new experiences and sensations, seeking autonomy, differentiation, and personal identity, without their self-regulating mechanisms having yet matured completely [26]. Therefore, a still immature empathy, assertiveness, magical thinking (“that will not happen to me”), and a certain tendency to impulsiveness, which is involved differently in health-risk behaviors [27,28,29,30], characterize adolescents who show a desire for experimentation and innovation, while seeking the approval of friends and schoolmates through the use of ICT. 

Thus, the search for autonomy and personal identity is related to emotionally vulnerable adolescence, which can lead to cyber violence [31]. In this process of resolving one’s own identity, self-esteem, or the evaluation one makes of one’s own personal characteristics, is accompanied by positive and negative thoughts, feelings, and emotions. Higher self-esteem has been associated with greater capacity for autonomous decisions and emotional autonomy [32]. For a creative response, adequate levels must exist of emotional self-regulation, tolerance to frustration, and strong concentration and motivation, in addition to adequate decision-making. These conditions are also favored in individuals with high self-esteem [33] and with higher EI [34]. 

A growing number of studies have explored the relationship between personality and self-esteem from the Big Five Factor Theory [35]. John et al. [36] summarized the five personality factors: (1) neuroticism, from emotional stability to negative affect (anxiety, nervousness, sadness, stress); (2) extraversion, coping with the social and material world energetically, that is, with sociability, activity, assertiveness, and positive affect; (3) agreeableness, including prosocial attitudes toward others, that is, altruism, tenderness, trustworthiness, and modesty; (4) conscientiousness, the ability to control impulses to direct behavior toward certain goals or objectives, that is, involving thinking before acting, following norms and rules, planning, organizing, and prioritizing tasks; and (5) openness to experience refers to flexibility, depth, ingenuity, and complexity that can characterize both the mental and experiential life of a person. In the meta-analysis by Judge et al. [37], high scores on neuroticism were correlated with low scores in self-esteem. Other studies found a positive relationship between self-esteem and the extraversion and conscientiousness factors, and a positive, although weak, relationship between self-esteem and the agreeableness and openness to experience factors [38]. According to Anderson et al. [39], feeling personal mastery (more freedom to resist situational influences and more ability to behave consistently with internal traits) was correlated positively with personality traits, such as extraversion, conscientiousness, openness to experience, and self-esteem, and negatively with neuroticism.

EI, which directly influences improvement of adolescent wellbeing [40], can also assist in coping with the risks associated with this evolutionary period [41]. Some authors have shown that emotional intelligence can buffer the effects of mood disorders by augmenting creativity [42]. Mayer et al. [43] described emotional intelligence as a skill some people have that enables them to complete the complex processing of emotions, and that serves as a guide for thinking and acting. With respect to the relationship between personality traits and emotional intelligence, in the study by Salguero et al. [44], with a large sample of adolescents, emotional attention was positively related to personality factors, although the effect size was small, and clarity and emotional repair showed weak positive correlations with extraversion, affability, conscientiousness, and openness to experience, and negative correlations with neuroticism. 

As our main objective was to progress in the study of the relationship between personality and creativity, we hypothesized, based on previous empirical findings demonstrating the existence of a relationship between personality traits and self-esteem, as well as between personality traits and creativity, that both variables (self-esteem and emotional intelligence) could mediate in this relationship. This finding would mean that assets or resources, such as self-esteem and emotional intelligence [45,46], could reinforce the development of certain personal traits (openness to experience, for example) which, as reflected in previous studies, develop creativity. The objectives and hypotheses of this study are described below in the order in which the statistical analyses for testing our hypothetical mediation model were conducted: 

(1) Analyze the relationships between the components of EI, self-esteem, personality, and self-expressive creativity in a sample of adolescents. We expected that self-expressive creativity would correlate positively with EI, self-esteem, and all the personality traits except neuroticism, with which it would correlate negatively. 

(2) Establish different personality profiles and determine whether statistically significant differences exist in self-expressive creativity, self-esteem, and EI. We expected to find different personality patterns and that these would be significantly related with the variables studied: those personality profiles with fewer high scores in neuroticism would score significantly higher in self-esteem and EI, as well as in self-expressive creativity. 

(3) Analyze the mediating role of variables susceptible to intervention (self-esteem and EI) in the relationship between personality and self-expressive creativity. We expected for both variables to provide uniqueness to the relationships between the various personality dimensions and self-expressive creativity. 

## 2. Materials and Methods 

### 2.1. Participants

The research was designed as a descriptive correlational cross-sectional study. The participants were selected by random sampling. The inclusion criterion was that the participants must be in high school, and exclusion criterion was not having any learning or language problems that could impede their understanding of the content of the questionnaires and answering coherently. For this study, we had a sample of 742 students in middle and high school (68.6% and 31.4%, respectively), who were residing in the province of Almería, Spain. The average age of the sample at the time of study was 15.63 years (SD = 1.24), in a range from 13 to 19 years. Almost half were boys (46.7%) and a little over half were girls (53.3%). Of the parents, 34.2% had a higher level of education, 52% medium, and 13.4% had a low level of education.

### 2.2. Instruments

#### 2.2.1. Self-Expressive Creativity

The Self-Expressive Creativity subscale of the Creative Behavior Questionnaire: Digital (CBQD) by Hoffmann et al. [24] is based on the everyday creativity measures by Ivcevic and Mayer [23]. The CBQD has three subscales: (1) digital creativity achievement, (2) school-based everyday creativity and (3) self-expressive digital creativity. Creative achievement is really anything someone has achieved that is accreditable (either because he was paid for it or was awarded for it, etc.). School-based everyday activity includes anything creative the students may have done at school or for homework. Finally, self-expressive creativity is other creative efforts, which generally do not receive the same outside validation (with respect to their creativity or that the person is outstanding in that area) as the creative achievement component. In this study, we focused on the Self-Expressive Digital Creativity subscale. The subscale is comprised of 10 items expressed on a Likert-type scale from 1 to 5, where 1 denotes “never” and 5 denotes “four or more times”. It is a self-report measure of digital creative behavior. Some examples of the items on this scale are: [Have you…] “Posted a personal blog (e.g., geared toward your friends and family)?”, “Posted a blog post about something that is your hobby or leisure activity (i.e., not related to your school or social life)?”, and “Started a new blog?” The CBQD has adequate reliability and validity [24]. In our sample of high school students, the ordinal α for self-expressive creativity was 0.85 and the ω coefficient was ω = 0.86.

#### 2.2.2. Big Five Inventory 

This questionnaire [36] is comprised of 44 items that evaluate the big five personality factors: extraversion (e.g., “is reserved”), agreeableness (e.g., “tends to find fault with others”), conscientiousness (e.g., “can be somewhat careless”), neuroticism (e.g., “is relaxed, handles stress well”), and openness to experience (e.g., “prefers work that is routine”), on a Likert-type scale from 1 “totally disagree” to 5 “totally agree”. In this study, the reliability values were: extraversion (ordinal α = 0.79, ω = 0.79), agreeableness (ordinal α = 0.65, ω = 0.66), conscientiousness (ordinal α = 0.77, ω = 0.77), neuroticism (ordinal α = 0.76, ω = 0.77), and openness to experience (ordinal α = 0.76, ω = 0.77).

#### 2.2.3. Rosenberg Self Esteem Scale (RSE) 

This scale [47] has 10 items that provide a global scale of personal self-esteem, understood as feelings of personal worth and respect for oneself, through statements like, “On the whole, I am satisfied with myself”, to which the adolescents must answer on a Likert scale of 1 to 4, where 1 indicates “strongly agree” and 4 indicates strongly disagree”. The ordinal α and ω coefficient found for our study sample were both 0.90.

#### 2.2.4. Expression, Management, and Emotion Recognition Evaluation Scale (TMMS-24) 

This scale [48] is based on the Trait Meta-Mood Scale (TMMS) by Salovey et al. [49]. It is a self-informed measure in which adolescents express their opinions of their emotional skills and abilities for expression, understanding, and regulation on a Likert-type scale from 1 (totally disagree) to 5 (totally agree). The TMMS-24 evaluates three dimensions of emotional intelligence, each of them with eight items: emotional attention (ordinal α = 0.91, ω = 0.91), clarity of feelings (ordinal α = 0.89, ω= 0.89), and emotional repair (ordinal α = 0.86, ω = 0.87). Emotional attention refers to the level of awareness of one’s own feelings and moods (e.g., “I pay close attention to feelings”), emotional clarity refers to the capacity for understanding one’s own emotional states (e.g., “My feelings are clear to me”), and emotional repair refers to the capacity for regulating feelings and emotional states (e.g., “Although I am sometimes sad, I am usually optimistic).

### 2.3. Procedure

Before data collection, the management teams at the schools were contacted and informed of the objectives of the study, as well as confidentiality during data processing. When they had agreed to participate, sessions were scheduled and two members of the research team went to the schools to administer the questionnaires. The students filled out the tests voluntarily, with their express permission, anonymously and individually. In all cases, the ethical standards of research were met with an informed consent sheet. The study was approved by the Bioethics Committee of the University of Almeria (Almeria, Spain).

### 2.4. Data Analysis

This study is quantitative, observational, and cross-sectional. First, to identify the relationships between the individual variables (EI, self-esteem, and personality) and self-expressive creativity, the correlations matrix was calculated with the Pearson’s correlation coefficient, and the corresponding descriptive statistics.

A two-stage cluster analysis was performed to identify groups of cases with different profiles by personality factors. Two-stage clustering is used to identify different patterns in a dataset, so that data within the same group or cluster tend to be similar, and different from other groups. The “determine automatically” option was applied to find the number of clusters. This procedure automatically determines the optimal number of clusters using the criteria specified, in this case, log-likelihood. We followed [50] for cluster quality interpretation. In our case, on a scale of −1, where all cases are in the centers of other clusters to none, to 1, where all cases are located in the center of their own clusters, and 0.4 is of sufficient quality, i.e., the data show tight clustering for the average silhouette coefficient. 

Once the groups or clusters were identified, we compared the means to determine the existence of significant differences between groups with respect to self-expressive creativity and self-esteem, using the Student’s *t*-test for independent samples and Cohen’s *d* [51] to identify the effect size. 

Then, simple mediation models were computed, and finally, a multiple mediation model with two mediators in series. For this, the SPSS macro (SPSS Inc., Chicago, IL, USA) for computation of mediation models [52] was used. Bootstrapping was used with coefficients estimated from 5000 bootstraps, specifically the bias-corrected bootstrap for confidence intervals. Instrument reliability was tested by estimating the internal consistency of the scores. (1) First, an exploratory factor analysis was conducted on the polychoric correlation matrix using FACTOR software [53]. The Kaufman and Rousseeuw data were computed under a criterion of parametric analysis with Promin rotation. (2) The ordinal α coefficient, which is based on the polychoric correlation analysis [54] and is therefore more suitable for calculating the reliability of ordinal or Likert-type scale answers [55], was calculated.

In addition, McDonald’s ω coefficient was estimated, following Ventura-León and Caycho [56].

## 3. Results

### 3.1. Adolescent Self-Expressive Creativity, EI, Self-Esteem, and Personality: Descriptive and Correlational Analyses

As shown in the correlation matrix (Table 1), self-expressive creativity was correlated positively with emotional attention (*r* = 0.18, *p* < 0.001), and with personality factors: Extraversion (*r* = 0.18, *p <* 0.001), neuroticism (*r* = 0.12, *p* < 0.001), and openness to expression (*r* = 0.24, *p* < 0.001).

**Figure 1:**
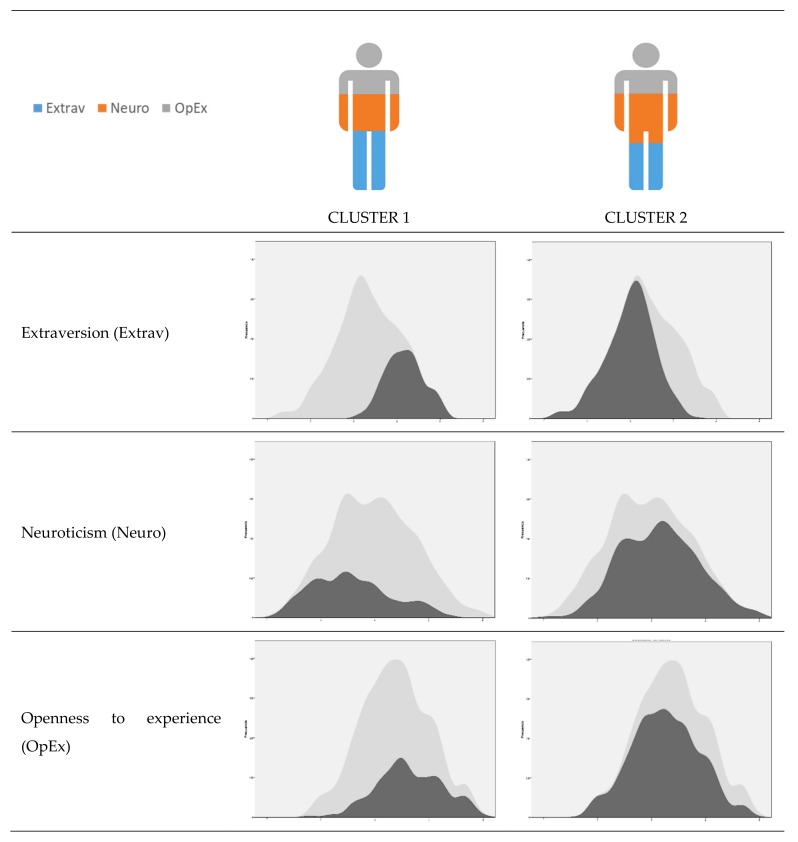
Profile by personality factors related to self-expressive creativity. Note: Factors are presented in order of importance of input. 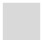 = total sample, 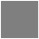 = subsamples (c1, c2). The vertical axis in figure indicates "frequency".

Based on the data found in the correlational analyses, associations were detected suggesting the existence of profiles based on the scores on some of the personality factors related to self-expressive creativity: extraversion, neuroticism, and openness to experience. Two profiles resulted from the inclusion of these variables in the cluster analysis (Figure 1), with the following distribution: 31.5% (*n* = 205) of the participants were in Cluster 1 and 68.5% (*n* = 445) in Cluster 2.

Cluster 1 was characterized by mean scores above those of the total sample for extraversion (*M* = 4.17) and openness to experience (*M* = 3.64), and neuroticism was below the total sample (*M* = 2.57). In Cluster 2, scores for extraversion (*M* = 2.99) and openness to experience (*M* = 3.25) were below the mean for the total sample, whereas the score for neuroticism was above the mean (*M* = 3.15).

After classification of groups, based on the two-cluster solution, a Student’s *t*-test for independent samples was calculated to identify any differences between the clusters with respect to the study variables: self-expressive creativity, self-esteem, and EI. First, significant differences were found between the clusters with respect to self-expressive creativity (*t* = 3.82, *p* < 0.001, *d* = 0.33), where the score for Cluster 1 (*M* = 2.23, *SD* = 0.65) was significantly higher than for Cluster 2 (*M* = 2.02, *SD* = 0.64). Mean scores for self-esteem in Cluster 1 (*M* = 33.31, *SD* = 6.32) were significantly higher (*t* = 7.83, *p* < 0.001, *d* = 0.68) than in Cluster 2 (*M* = 29.25, *SD* = 6.12). The EI components were observed to be statistically significantly different in emotional clarity (*t* = 7.57, *p* < 0.000, *d* = 0.64), with a higher mean score in Cluster 1 (*M* = 27.60, *SD* = 7.22) than in Cluster 2 (*M* = 22.94, *SD* = 6.96). For emotional repair, Cluster 1 (*M* = 29.27, *SD* = 7.01) had a significantly higher score (*t* = 7.68, *p* < 0.001, *d* = 0.65) than Cluster 2 (*M* = 24.56, *SD* = 7.07).

### 3.2. Mediation Models for Self-Esteem and EI on the Relationship of Personality and Self-Expressive Creativity

Based on the results from the analyses above, we needed to analyze the mediational relationship between the variables (Table 2). Model 1 examined intervention of self-esteem as a possible mediator in the effect of personality on self-expressive creativity (coefficient of determination (*R^2^*) = 0.086). In this case, the data extracted were for a model in which personality and self-esteem were statistically significant (*B* = −4.06, *p* < 0.001), but no direct effect of self-esteem on self-expressive creativity was found (*B* = −0.003, *p* = 0.46).

In Model 2, emotional attention was proposed as the mediator in the effect of personality on self-expressive creativity (*R^2^* = 0.063). The effect of personality on emotional attention was not statistically significant (*B* = 0.41, *p* = 0.54). However, emotional attention was directly related to self-expressive reativity (*B* = 0.01, *p* < 0.001).

Emotional clarity was entered in Model 3 as a possible mediator in the personality–self-expressive creativity relationship (*R^2^* = 0.087). A significant relationship was found of personality on the mediator (*B* = −4.66, *p* < 0.001), but without effect on self-expressive creativity (*B* = 0.000, *p* = 0.93).

The third component of EI, repair, was included as the mediator in Model 4 (*R^2^* = 0.086), where a significant effect of personality on it was observed (*B* = −4.65, *p* < 0.001), but no effect of emotional repair was observed on self-expressive creativity (*B* = 0.000, *p* = 0.82).

**Figure 2:**
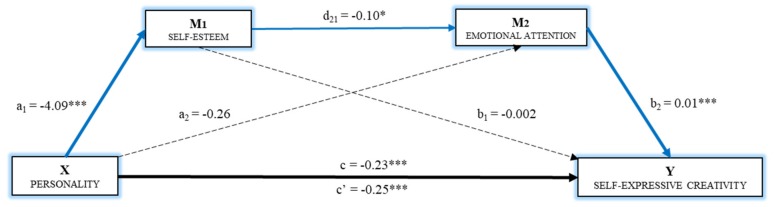
Multiple mediation model of self-esteem and emotional attention on the relationship between personality and self-expressive creativity (Note. * *p* < 0.05, *** *p* < 0.001. Non-standardized regression coefficients).

Finally, based on the relationships established by the simple mediation models above, Model 5 proposed a multiple mediation analysis with two mediator variables forming a causal chain (*R^2^* = 0.091). Thus, both self-esteem and emotional attention were entered in the model as Mediator 1 and Mediator 2, respectively (Figure 2).

First, the effect of the independent variable on the two mediators was estimated, where a significant effect was observed on self-esteem (*B* = −4.09, *p* < 0.001), but not on emotional attention (*B* = –0.26, *p* = 0.72]. The direct effect of personality on self-expressive creativity was significant (*B* = −0.25, *p* < 0.001).

Finally, based on the analysis of the indirect effects by bootstrapping, data were found that support a significant level for Path 2 (Indirect (Ind)2: X→ Model 1 (M1)→Model 2 (M2)→Y; B = 0.006, SE = 0.004, 95% CI (0.000, 0.018)). Thus, belonging to personality Cluster 2 (adolescents with scores below the mean in extraversion and openness to experience, and scores over the mean in neuroticism) had a negative effect on self-esteem, which, in turn, had a negative effect on emotional attention, with a significant effect on self-expressive creativity. That is, belonging to a personality cluster characterized by higher scores in neuroticism and lower in extraversion and openness to experience had a direct negative effect on self-expressive creativity, in addition to an indirect effect, through self-esteem and emotional attention, where these act as mediators operating in series.

## 4. Discussion

We discuss the main themes of this study in the order in which the results described above were addressed. First, the correlational analysis between self-expressive creativity and the components of EI showed that self-expressive creativity and EI are related to each other. Thus, the higher the scores of the adolescents in self-expressive creativity (referring to behaviors that express identity and personal interests, handcraft activities, and social expression), the greater their ability to recognize their feelings and know what they mean. Although little previous empirical evidence exists concerning the relationship between digital creativity and EI, some studies have analyzed the relationship between creativity and EI from another perspective and with other population groups finding similar results [24]. 

Concerning the relationship between self-expressive creativity and the various dimensions of personality, self-expressive creativity was positively associated with extraversion, openness to experience, and neuroticism. Thus, high scores by students in self-expressive creativity were associated with more energy or enthusiasm, more ingenuity or mental openness, as well as with negative affect or nervousness. Similar results were found in the study by Ivcevic [10] on everyday creativity, which was correlated significantly and positively with personality traits such as extraversion and conscientiousness, and in the study by Hoffmann et al. [14], in which self-expressive creativity in the digital domain was positively associated with openness to experience. In the study by Silvia et al. [11], the time devoted to everyday creative activities was found to be positively correlated with openness to experience and conscientiousness, and negatively with neuroticism. According to Ivcevic [10], whereas artistic creativity is positively related to psychopathology, the same is not true of everyday creativity, which is correlated with personal growth. Our later analyses, which are discussed below, suggest the same.

Second, with regard to the relationship between self-esteem and personality, adolescents with higher self-esteem also scored higher in extraversion (energy or enthusiasm) and conscientiousness (control or restriction), with a moderate effect size; agreeableness (altruism or affect), with a small effect size; and lower scores in neuroticism (negative affect or nervousness), with a moderate effect size. These results are coherent with those reported by Judge et al. [27], who found significant negative correlations between neuroticism and self-esteem, with an even higher effect size than in our study. The results of the study by Robins et al. [28] were similar for the correlations between self-esteem and the extraversion, conscientiousness, and agreeableness dimensions. In their case, they also found a significantly positive but weak association between openness to experience and self-esteem.

Third, we found different adolescent personality profiles. The cluster analysis enabled us to identify a first group (31.5% of the sample) composed of adolescents who scored high in extraversion and openness to experience and low in neuroticism, and a second group (68.5% of the sample) composed of adolescents who scored low in extraversion and openness to experience, and high in neuroticism. The first group of adolescents had higher scores in self-expressive creativity, self-esteem, clarity, and emotional repair (understanding and regulating mental states adequately) than the second group. These empirical findings are coherent with those reported in other studies that suggested the same with regard to the relationship between personality and self-expressive creativity [12,14], personality and self-esteem [27], and personality and EI [34]. Our data showed a stronger relationship between personality characteristics and EI than reported by Salguero et al. [34], perhaps because our analysis included three significant dimensions (extraversion, openness to experience, and neuroticism) in the personality profiles, instead of analyzing each of the personality traits and their relationship with EI independently. Our data also mainly support the relationship between the personality profiles found and the dimensions of understanding and emotional regulation, without considering emotional attention, which, in that study, was moderately negatively correlated with neuroticism. Therefore, although our mediation study provides a first approximation, future studies could continue analyzing the role of EI in the development of other personality traits (such as neuroticism). 

Finally, another of the objectives of this study was to analyze the relationship between self-expressive creativity and personality in greater depth based on the mediating effect of variables susceptible to intervention, such as self-esteem and EI. The results showed that being part of the second group of adolescents had a negative effect on self-esteem, which in turn had a negative repercussion on emotional attention, with a direct negative effect on self-expressive creativity. In other words, belonging to Group 2, where the scores in neuroticism were high and low in extraversion and openness to experience, had a direct negative effect on self-expressive creativity and an indirect negative effect through self-esteem and emotional attention. Therefore, adolescents in the second group showed less self-expressive creativity due to their low self-esteem, which negatively influenced their awareness of their own emotions, that is, emotional attention or the ability to recognize feelings, knowing what they mean, and expressing them adequately. Our findings also demonstrate the relevant role of self-esteem in emotional attention, associated in other studies with high scores in neuroticism [34], and its effect on self-expressive creativity. Other studies have not found a direct relationship between EI and creative behavior either, finding that openness to experience was the personality component that favored creativity to the greatest extent [12]. Other authors have suggested that EI rather buffered the effects of mood disorders, increasing creativity [32].

### 4.1. Limitations

These findings are novel and relevant to the field of everyday creativity, and in particular, with respect to self-expressive creativity in the digital domain. However, the cross-sectional study design does not permit us to establish causal relationships between the variables analyzed, so new studies should be conducted to replicate these results with samples of adolescents from different countries and cultures, as well as longitudinal studies that also corroborate the findings of this cross-sectional design, contributing to the generalization of the results, and examining the role of emotions in digital creativity. One other limitation of this study is related to the evaluation of digital creativity, for which a self-report measure was chosen, which may have included desirability and social acceptability biases. However, similar equally valid and reliable previous experiments have also employed self-report scales to evaluate everyday creative behavior [13]. We further suggest future studies employ other measures including tasks evaluating different domains of creativity along with these procedures.

### 4.2. Implications

This study has clear practical implications. On one hand, it demonstrated that access to the Internet can be used positively to reinforce self-expressive creativity. On the other, the relationship between certain personality profiles and self-expressive creativity, self-esteem, and emotional intelligence shows how digital self-expressive creativity develops in both formal and informal education, and contributes to appropriate use of the new technologies. 

## 5. Conclusions

Based on these results, a plan should be developed for positive adolescent use of the Internet that reduces cyberbullying, with a creative model enabling digital self-expression for acquiring identity and self-efficacy through the positive influence of peers and promoting feelings of personal mastery and self-affirmation through constructive tasks reinforcing self-esteem and EI. Thus, research should continue on the relationships between these aspects, from the development of self-efficacy, self-esteem, and feelings of personal mastery, to resist negative environmental influences and to create behavior more consistent with internal traits, avoiding cyberbullying, and assist with increasing self-esteem and emotional conscientiousness. In our study, personality variables, such as extraversion, openness to experience, and low neuroticism, were found to be essential to self-expressive creativity in the digital domain.

## Figures and Tables

**Table 1:** Correlations and descriptive statistics: self-expressive digital creativity, emotional intelligence (EI), self-esteem, and personality.

	1	2	3	4	5	6	7	8	9	10
1. Self-expressive creativity	–									
2. Emotional attention	0.18^***^	–								
3. Emotional clarity	0.03	0.18^***^	–							
4. Emotional repair	0.03	0.13^**^	0.38^***^	–						
5. Self-esteem	0.02	−0.05	0.31^***^	0.33^***^	–					
6. Extraversion	0.18^***^	0.08^*^	0.28^***^	0.26^***^	0.31^***^	–				
7. Agreeableness	−0.02	0.04	0.20^***^	0.33^***^	0.22^***^	0.16^***^	–			
8. Conscientiousness	0.02	0.06	0.16^***^	0.26^***^	0.22^***^	0.11^**^	0.28^***^	–		
9. Neuroticism	0.12^**^	0.30^***^	−0.30^***^	−0.36^***^	−0.34^***^	−0.12^**^	−0.35^***^	−0.18^***^	–	
10. Openness to experience	0.24^***^	0.15^***^	0.11^**^	0.20^***^	0.06	0.14^***^	0.08^*^	0.15^***^	−0.01	–
Mean	2.08	25.69	24.40	25.96	30.49	3.35	3.64	3.24	2.96	3.37
SD	0.66	7.66	7.32	7.41	6.42	0.76	0.56	0.70	0.75	0.63

Note. * *p* < 0.05, ** *p* < 0.01, *** *p* < 0.001.

**Table 2:** Mediation models for self-esteem and the EI components in the relationship between personality and self-expressive creativity.

**Model 1 (M1): PERS → SE → SE-C**		B	SE	*t*	95% CI
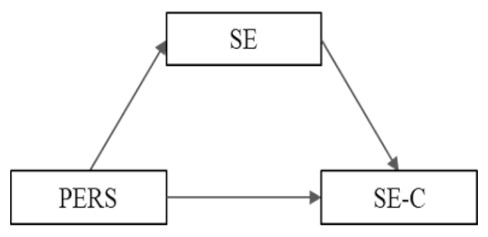	PERS → SE	−4.068 ^***^	0.559	−7.269	(−5.167, −2.969)
	SE → SE-C	−0.003	0.004	−0.734	(−0.011, 0.005)
	Total effect PERS → SE-C	−0.236 ^***^	0.057	−4.083	(−0.350, −0.122)
	Direct effect PERS → SE-C	−0.249 ^***^	0.060	−4.117	(−0.368, −0.130)
	Indirect effect PERS → SE-C	0.013	0.019		(−0.025, 0.051)
**Model 2 (M2):** PERS → EM-ATT → SE-C		B	SE	*t*	95% CI
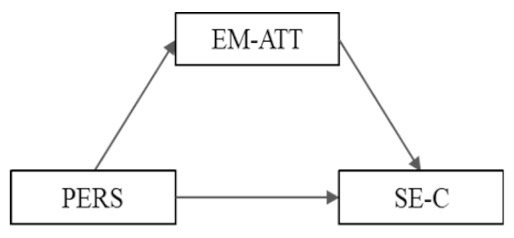	PERS → EM-ATT	0.415	0.678	0.612	(−0.917, 1.748)
	EM-ATT → SE-C	0.016 ^***^	0.003	4.914	(0.009, 0.023)
	Total effect PERS → SE-C	−0.213 ^***^	0.056	−3.812	(−0.324, −0.103)
	Direct effect PERS → SE-C	−0.220 ^***^	0.055	−4.010	(−0.328, −0.112)
	Indirect effect PERS → SE-C	0.006	0.011		(−0.013, 0.031)
**Model 3 (M3):** PERS → EM-CLA → SE-C		B	SE	*t*	95% CI
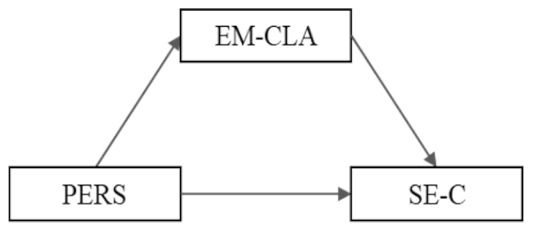	PERS → EM-CLA	−4.667 ^***^	0.629	−7.416	(−5.903, −3.431)
	EM-CLA → SE-C	−0.000	0.003	−0.082	(−0.007, 0.007)
	Total effect PERS → SE-C	−0.189 ^**^	0.057	−3.300	(−0.301, −0.076)
	Direct effect PERS → SE-C	−0.190 ^**^	0.060	−3.174	(−0.308, −0.072)
	Indirect effect PERS → SE-C	0.001	0.018		(−0.034, 0.038)
**Model 4 (M4):** PERS → EM-REP → SE-C		B	SE	*t*	95% CI
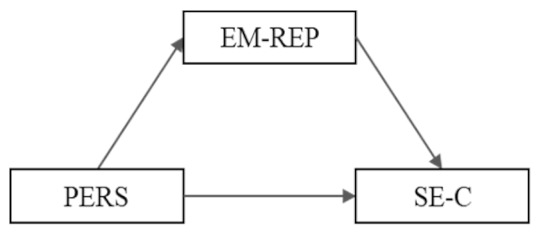	PERS → EM-REP	−4.650 ^***^	0.624	−7.441	(−5.877, −3.422)
	EM-REP → SE-C	−0.000	0.003	−0.216	(−0.008, 0.006)
	Total effect PERS → SE-C	−0.217 ^***^	0.057	−3.767	(−0.330, −0.103)
	Direct effect PERS → SE-C	−0.220 ^***^	0.060	−3.661	(−0.339, −0.102)
	Indirect effect PERS → SE-C	0.003	0.017		(−0.031, 0.037)
**Model 5 (M5):** PERS → SE → EM-ATT → SE-C		B	SE	*t*	95% CI
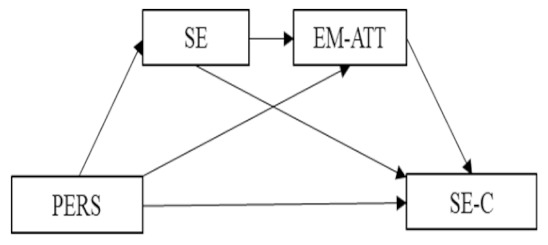	Total effect PERS → SE-C	−0.236 ^***^	0.058	−4.055	(−0.350, −0.121)
	Direct effect PERS → SE-C	−0.250 ^***^	0.060	−4.168	(−0.358, −0.132)
	Ind 1: PERS → SE → SE-C	0.011	0.020		(−0.027, 0.053)
	Ind 2: PERS → SE → EM-ATT → SE-C	0.006	0.020		(0.000, 0.018)
	Ind 3: PERS → EM-ATT → SEC-C	−0.003	0.004		(−0.028, 0.016)

Note. PERS = Personality, SE = Self-esteem, SE-C = Self-expressive creativity, EM-ATT = Emotional attention, EM-CLA = Emotional clarity, EM-REP = Emotional repair; Ind = indirect effect, SE = Standard Error, CI = Confidence interval, *B* = non-standardized regression coefficient; ** *p* < 0.01, *** *p* < 0.001.

## References

[1] Smith P.K., Mahdavi J., Carvalho M., Fisher S., Russell S., Tippett N. (2008). Cyberbullying: Its nature and impact in secondary school pupils. J. Child Psychol. Psiquiatr..

[2] Lee Y.C., Wu W.-L. (2018). Factors in cyber bullying: The attitude-social influence-efficacy model. Ann. Psicol..

[3] Van Goethem A.A., Scholte R.H., Wiers R.W. (2010). Explicit and implicit bullying attitudes in relation to bullying behavior. J. Abnorm. Child Psychol..

[4] Avilés J.M., Petta R. (2018). The Peer Support Systems for the promotion of convivencia, the improvement of the classroom climate and the prevention of situations of bullying: The experience of Brazil and Spain. Eur. J. Dev. Educ. Psychop..

[5] Carbonell N., Cerezo F. (2019). El programa cie: Intervención en ciberacoso escolar mediante el desarrollo de la inteligencia emocional [CIE program: Intervention in cyberbullying by de development of the Emotional Intelligence]. Eur. J. Health. Res..

[6] Beltrán-Catalán M., Zych I., Ortega-Ruiz R., Llorent V.J. (2018). Victimisation through bullying and cyberbullying: Emotional intelligence, severity of victimisation and technology use in different types of victims. Psicothema.

[7] Gázquez J.J., Pérez-Fuentes M.C., Carrión J.J., Santiuste V. (2010). Estudio y análisis de conductas violentas en Educación Secundaria en España [Study and analysis of violent behaviour in Secondary Education in Spain]. Univ. Psychol..

[8] Muros B., Aragón Y., Bustos A. (2013). La ocupación del tiempo libre de jóvenes en el uso de videojuegos y redes [Youth’s usage of leisure time with video games and social networks]. Comunicar.

[9] Cheung C.M., Chiu P.Y., Lee M.K. (2011). Online social networks: Why do students use facebook?. Comput. Hum. Behav..

[10] Guilford J.P. (1950). Creativity. Am. Psychol..

[11] Torrance E., Sterrnberg R.J. (1988). The nature of creativity as manifest in its testing. The Nature of Creativity: Contemporary Psychological Perspectives.

[12] Amabile T.M. (1983). The social psycholoy of creativity: A componential conceptualization. J. Personal. Soc. Psychol..

[13] Csikszentmihalyi M. (1996). Creativity. Flow and the Psychology of Discovery and Invention.

[14] Sternberg R.J., Lubart T.I. (1996). Invest in creativity. Am. Psychol..

[15] De Bono E. (1992). Serious Creativity: Using the Power of Lateral Thinking to Create New Ideas.

[16] Amabile T.M. (1985). Motivation and creativity: Effects of motivational orientation creative writers. J. Personal. Soc. Psychol..

[17] Gardner H. (1993). Creating Minds.

[18] Richards R., Kinney D.K., Benet M., Merzel A.P.C. (1988). Assessing everyday creativity: Characteristics of the Everyday Creativity Scales and validation with three large samples. J. Personal. Soc. Psychol..

[19] Runco M.A., Sternberg R.J., Grigorenko E.L., Singer J.L. (2004). Everyone has creative potential. Creativity: From Potential to Realization.

[20] Ivcevic Z. (2007). Artistic and everyday creativity: An act-frequency approach. J. Creat. Behav..

[21] Silvia P.J., Beaty R.E., Nusbaum E.C., Eddington K.M., Levin-Aspenson H., Kwapil T.R. (2014). Everyday creativity in daily life: An experience-sampling study of “little c” creativity. Psychol. Aesthet. Creat. Arts.

[22] Ivcevic Z., Brackett M.A., Mayer J.D. (2007). Emotional intelligence and emotional creativity. J. Personal..

[23] Ivcevic Z., Mayer J.D. (2009). Mapping dimensions of creativity in the life-space. Creat. Res. J..

[24] Hoffmann J., Ivcevic Z., Brackett M. (2016). Creativity in the age of technology: Measuring the digital creativity of millennials. Creat. Res. J..

[25] Wechsler S.M., Benson N., de Lara W., Bachert M.D.A., Gums E.F. (2018). Adult temperament styles: A network analysis of their relationships with the Big Five Personality Model. Eur. J. Educ. Psychol..

[26] Oliva A. (2012). Desarrollo cerebral y asunción de riesgos durante la adolescencia [Brain development and risk taking during adolescence]. Apunt. Psicol..

[27] Martínez-Loredo V., Fernández-Hermida J.R., de La Torre-Luque A., Fernández-Artamendi S. (2018). Polydrug use trajectories and differences in impulsivity among adolescents. Int. J. Clin. Health.

[28] Nieto B., Portela I., López E., Domínguez V. (2018). Verbal violence in students of compulsory secondary education. Eur. J. Investig..

[29] Pérez-Fuentes M.C., Molero M.M., Barragán A.B., Gázquez J.J. (2019). Profiles of violence and alcohol and tobacco use in relation to impulsivity: Sustainable consumption in adolescents. Sustainability.

[30] Pérez-Fuentes M., Gázquez J.J., Molero M.M., Cardila F., Martos A., Barragan A.B., Garzón A., Carrión J.J., Mercader I. (2015). Adolescent impulsiveness and use of alcohol and tobacco. Eur. J. Investig..

[31] Ortega R., Elipe P., Mora-Merchán J.A., Genta M.L., Brighi A., Guarini A., Smith P.K., Thompson F., Tippett N. (2012). The emotional impact of bullying and cyberbullying on victims: A European cross-national study. Aggress. Behav..

[32] Alonso-Stuyck P., Zacarés J.J., Ferreres A. (2018). Emotional separation, autonomy in decision-making, and psychosocial adjustment in adolescence: A proposed typology. J. Child Fam. Stud..

[33] Wang Y., Wang L. (2016). Self-construal and creativity: The moderator effect of self-esteem. Personal. Individ. Differ..

[34] Nori R., Signore S., Bonifacci P. (2018). Creativity style and achievements: An investigation on the role of emotional competence, individual differences and psychometric intelligence. Front. Psychol..

[35] Erol R.Y., Orth U. (2011). Self-esteem development from age 14 to 30 years: A longitudinal study. J. Personal. Soc. Psychol..

[36] John O.P., Donahue E.M., Kentle R.L. (1991). The Big Five Inventory-Versions 4a and 54.

[37] Judge T.A., Erez A., Bono J.E., Thoresen C.J. (2002). Are measures of selfesteem, neuroticism, locus of control, and generalized self-efficacy indicators of a common core construct?. J. Personal. Soc. Psychol..

[38] Robins R.W., Hendin H.M., Trzesniewski K.H. (2001). Measuring global self-esteem: Construct validation of a single-item measure and the Rosenberg Self-Esteem Scale. Personal. Soc. Psychol. Bull..

[39] Anderson C., John O.P., Keltner D. (2012). The personal sense of power. J. Personal..

[40] Molero M.M., Pérez-Fuentes M.C., Barragán A.B., del Pino R.M., Gázquez J.J. (2019). Analysis of the Relationship between Emotional Intelligence, Resilience, and Family Functioning in Adolescents’ Sustainable Use of Alcohol and Tobacco. Sustainability.

[41] Prado V., Villanueva L., Górriz A.B. (2018). Trait emotional intelligence and subjective well-being in adolescents: The moderating role of feelings. Psicothema.

[42] Guastello S.J., Guastello D.D., Hanson C.A. (2004). Creativity, mood disorders, and emotional intelligence. J. Creat. Behav..

[43] Mayer J.D., Salovey P., Caruso D.R. (2008). Emotional intelligence: New ability or eclectic traits?. Am. Psychol..

[44] Salguero J.M., Fernandez-Berrocal P., Balluerka N., Aritzeta A. (2010). Measuring perceived emotional intelligence in the adolescent population: Psychometric properties of the Trait Meta-Mood Scale. Soc. Behav. Personal..

[45] Seligman M. (2003). Positive Psychology: Fundamental assumptions. Psychologist.

[46] Seligman M., Csikszentmihalyi M. (2000). Positive Psychology: An introduction. Am. Psychol..

[47] Rosenberg M. (1965). Society and the Adolescent Self-Image.

[48] Fernández-Berrocal P., Extremera N., Ramos N. (2004). Validity and reliability of the Spanish modified version of the Trait Meta-Mood Scale. Psychol. Rep..

[49] Salovey P., Mayer J.D., Goldman S.L., Turvey C., Palfai T.P., Pennebaker J.W. (1995). Emotional attention, clarity and repair: Exploring emotional intelligence using the trait meta-mood scale. Emotion, Disclousure and Health.

[50] Kaufman L., Rousseeuw P.J. (1990). Finding Groups in Data. An Introduction to Cluster Analysis.

[51] Cohen J. (2013). Statistical Power Analysis for the Behavioral Sciences.

[52] Hayes A.F. (2013). Introduction to Mediation, Moderation, and Conditional Process Analysis: A Regression-Based Approach.

[53] Lorenzo-Seva U., Ferrando O.J. (2006). FACTOR: A Computer program to fit the exploratory factor analysis model. Behav. Res. Methods.

[54] Domínguez-Lara S. (2018). Fiabilidad y alfa ordinal [Reliability and ordinal alpha]. Actas Urol. Españolas.

[55] Elosua P., Zumbo B.D. (2008). Coeficientes de fiabilidad para escalas de respuesta categórica ordenada [Reliability coefficients for ordinal response scales]. Psicothema.

[56] Ventura-León J.L., Caycho T. (2017). El coeficiente Omega: Un método alternativo para la estimación de la confiabilidad [The Omega coefficient: An alternative method for estimating reliability]. Rev. Latinoam. Cienc. Soc. Niñez Y Juv..

